# The effect of conscious mindfulness-based informative approaches on managing symptoms in hemodialysis patients

**DOI:** 10.3389/fpsyg.2024.1363769

**Published:** 2024-05-09

**Authors:** Gönül Akbulut, Behice Erci

**Affiliations:** ^1^Aşkale Vocational School, Atatürk University, Erzurum, Turkey; ^2^Faculty of Nursing, Inönü University, Malatya, Turkey

**Keywords:** conscious mindfulness, hemodialysis, awareness, symptom, nursing, complementary medicine, alternative medicine

## Abstract

**Introduction:**

The research was conducted to determine the effect of conscious mindfulness based informative approaches applied in hemodialysis patients on reducing stress and managing symptoms.

**Methods:**

This research was conducted as a real experimental model with a control group. Research population consisted of 160 hemodialysis patients. The sample of the study was determined as 120 hemodialysis patients in total, 60 in the experimental and 60 in the control group, as a result of the power analysis. After the pre-test application, a mindfulness-based stress reduction program was applied to the experimental group. In the analysis of the data collected in the research, percentage, frequency, chi-square analysis, *t*-test for independent groups, *t*-test for dependent groups were used by means of SPSS for Windows 22.00 statistical software package.

**Results:**

The *t*-test analyses of the differences between pre-test and post-test scores of hemodialysis patients in the experimental group were found to be significant in favor of the post-tests.

**Discussion:**

It was found out that the conscious mindfulness-based informative approaches decreased the perceived stress and anxiety of the patients in the experimental group, whereas increased their levels of conscious mindfulness and symptom management.

## 1 Introduction

Administration of hemodialysis (HD) treatment has helped the lengthening of the patients’ lifetime, as a result of which development all attention has been directed to the problems experienced by hemodialysis patients (Sanlıtürk et al., 2018). It is observed that HD patients have been suffering itching and rashes, skin dryness, fatigue, nausea, vomiting, constipation, diarrhea, sleep disorders, pain, muscle cramps, sexual problems, body image disturbance, impaired social functioning, problems in family/business/school life, economic difficulties, burnout, stress, depression, and anxiety (Almutary, 2022). Rate, frequency and intensity of symptoms vary from one patient to another, however, as the intensity and frequency of the symptoms get higher, many of the patients exhibit despair, ambiguity, burnout and depression, resulting in impaired quality of life (Tolasa and Akyol, 2017).

According to Almutary’s (2022) study, patients undergoing hemodialysis develop high levels of depression and daytime somnolence, which symptoms appear to be the main determinant of life quality. Another study undertaken suggests that patients undergoing hemodialysis have been frequently suffering anxiety and depression, which symptoms exert adverse impacts on the patients’ quality of life. According to the same research, positive effects are achieved when symptoms of anxiety and depression are determined and treated at an early stage (Al-Nashri and Almutary, 2022).

According to Eren’s (2019) research, most encountered symptoms in hemodialysis patients are fatigue or lethargy, reduced sexual interest, reduced sexual satisfaction, and nervous temperament, whereas least frequent ones are muscle pain, difficulty in keeping the legs still, and diarrhea. In the study by Akgöz and Arslan (2017), it has been identified that most frequent symptoms in HD patients were fatigue, reduced physical energy, headache, bone pain and arthralgia.

In conclusion in the case of HD patients, developing terminal behaviors in lifestyle, achieving adherence to therapy, and controlling symptoms are of major importance. Health professionals who have sufficient training and knowledge on HD treatment should assume significant roles such as informing the patients about the treatment they have been receiving and providing them with necessary knowledge and skills (Al-Nashri and Almutary, 2022). However, according to many researches, it has been observed that individual awareness of hemodialysis patients is quite low despite all suggestions made and guides drawn up in that regard (Tuot et al., 2015).

Conscious mindfulness (CM) is a concept that is related to focusing on the present moment, which involves observations and acceptance without judgment on inner and outer experiences (Mantzios and Giannou, 2018). CM suggests that people may, by staying in the present moment, cope with emotional distress which comes along with their thoughts (Igarashi et al., 2021). When the level of awareness in respect of what is being experienced in the present moment is low, automatic behaviors not controlled by the conscious are displayed. As CM level of the individuals gets higher, they develop stronger coping strategies in adverse conditions (Tırıskan et al., 2015). CM-based approaches may be used in helping the individual patients to become aware of their potential power, establish body-mind connection, and in accelerating the healing process. Furthermore, CM-based approaches serve as an effective tool in regard to strengthening the communication between the health professional and the patient, and also in terms of creating a suitable therapeutic environment (Körükcü and Kukulu, 2015).

Various methods are being applied to raise the level of awareness. One of these methods is the mindfulness-based stress reduction program (MBSRP). Kabat-Zinn (2003) has conducted the earliest studies on this subject at the Mindfulness Center of the Massachusetts Institute of Technology and founded the mindfulness-based stress reduction program (MBSRP). MBSRP was initially formed to reduce the pains of patients but later used to cope with problems such as stress as well. MBSRP is a group program that proceeds step by step, which is applicable as an individual method or as a complementary, supportive one (Catak and Ogel, 2010; Gherardi-Donato et al., 2023).

MBSRP is being applied in treatment of symptoms associated with cancer, treatment of obesity, treatment of sleep disorders, functional healing of schizophrenic patients, symptomatic treatment of Parkinson patients, and reduction of anxiety in transplant recipients, along with increasing the level of motivation and conscious mindfulness in non-patient groups (Harvard Business School Publishing and Corporation, 2017). MBSRP helps people to develop acceptance and alternative reactions instead of negative thought (Ji et al., 2023). CM training is related to changes affecting biological processes, such as heartbeat, breathing and immune functions, in brain regions that are responsible for stress reactions and emotional regulation (Boyd et al., 2018).

Studies undertaken have shown that, in the case of HD patients displaying symptoms of anxiety and depression, MBSRP is an applicable and tolerable program that might yield positive results (Thomas et al., 2017).

It has been scientifically proven that MBSRP enhances the quality of life and self-sufficiency/efficacy levels of HD patients (Solati et al., 2019), reduces anxiety and depression (Haghshenas et al., 2019), and improves general mental health and biochemical marker levels of individuals (Sohn et al., 2018). Moreover, MBSRP reduces physical symptoms, anxiety, sleep disorders, social malfunctions, depression symptoms (Nejad et al., 2018), and improves self-compassion and serum phosphorus levels (Igarashi et al., 2021).

In conclusion this research has been conducted with a view to determining the effect of conscious mindfulness-based informative approaches applied in hemodialysis patients on reducing stress and managing symptoms.

To achieve the purpose of the research, the following hypotheses were tested.

Hypothesis 1: Mindfulness-based informative approaches reduce stress levels in hemodialysis patients.

Hypothesis 2: Mindfulness-based informative approaches provide symptom management in hemodialysis patients.

## 2 Materials and methods

This research was conducted as a real experimental model with a control group.

### 2.1 Participants

Research population consisted of 160 dialysis patients who were registered in Atatürk University Research Hospital Hemodialysis Unit and Health Sciences University Erzurum Regional Training and Research Hospital Hemodialysis Unit. The sample of the study was determined as 120 hemodialysis patients in total, 60 in the experimental and 60 in the control group, as a result of the power analysis with 0.05 margin of error, 0.95 confidence interval, 0.6 effect size, and 0.98 representation power. Randomization method was employed in the selection and grouping of samples. The sample was created by taking into account the inclusion and exclusion criteria for the study. The sample was made up of individuals over the age of 18 who were open to communication and agreed to participate in the study (Mertens, 2019). Throughout the research process no patients have left the experimental or control group wherefore the research was completed with the participation of 120 patients who agreed to take part in the study. In order to prevent patients from being affected by each other during the research process, drawing of lots between two dialysis centers has taken place as a result of which the patients hospitalized at Atatürk University Research Hospital Hemodialysis Unit were determined as the experimental group.

### 2.2 Measure

In the gathering of data, personal information sheets, mindful attention awareness scale, dialysis symptom index, perceived stress scale, and Beck anxiety scale were used.

#### 2.2.1 Personal data collection form

This personal information sheet is a questionnaire prepared by the researchers, which includes socio-demographic details (age of patient, level of education, spouse’s level of education, employment status, spouse’s employment status, economic condition) and HD-related data.

#### 2.2.2 Mindful attention awareness scale

Mindful attention awareness scale (MAAS) was developed by Brown and Ryan (2003). Adaptation efforts respecting its validity and reliability in the Turkish domain were undertaken by Ozyeşil et al. (2011). MAAS which consists of 15 items is a scale that measures the overall tendency with regard to receptive awareness of and attention to momentary experiences in daily life. MAAS has a one-factor structure and gives a single total score. High scores obtained from the scale indicate high levels of mindfulness. MAAS is a 6-level (almost always, usually, sometimes, quite seldom, rarely, almost never) Likert type scale. Cronbach Alpha coefficient of the scale is 0.82. In this study, subject coefficient was found as 0.90.

#### 2.2.3 Dialysis symptom index

Dialysis symptom index (DSI) was developed by Weisbord et al. (2004) with a view to measuring the level of distress experienced by hemodialysis patients in respect of symptoms. Adaptation efforts respecting its validity and reliability in the Turkish domain were undertaken by Onsöz and Yesilbalkan (2013). Answers regarding symptoms experienced in the last seven days are given as “yes” or “no.” When the answer is “yes,” a 5-level Likert scale, i.e., 1 = none, 2 = a little, 3 = sometimes, 4 = very little, 5 = too much, is applied to determine to what degree the patient’s life is affected by the symptom in question. Points obtained are added to find the total scale score. Total score from the index may vary between 0 and 150. No symptoms are present if the score is 0. Where the total score has approached 150, such situation indicates that the effect of subject symptom has increased. Cronbach Alpha coefficient of the scale is 0.89. On the other hand, cited coefficient was found as 0.87 in this research.

#### 2.2.4 Perceived stress scale

Perceived stress scale (PSS) was developed by Cohen, Kamarck and Mermelstein. Adaptation efforts respecting its validity and reliability in the Turkish domain were undertaken by Eskin et al., 2013. PSS is a 14-item scale that has been designed to measure how stressful certain situations in the individual’s life are perceived. Participants evaluate every item on a 5-level Likert type scale which ranges between “Never (0)” and “Very frequently (4).” 7 of the items which involve positive statements are given points in the reverse order. PSS forms have 14, 10 and 4-item versions. Cronbach Alpha coefficient of the scale is 0.86. In this study, such coefficient was found as 0.80.

#### 2.2.5 Beck anxiety scale

Beck anxiety scale (BAS) was developed by Beck et al. (1988) to evaluate the frequency of anxiety symptoms. Adaptation work respecting its validity and reliability in our country was undertaken by Ulusoy et al. (1998) BAS which consists of twenty-one items is a Likert type evaluation scale with a scoring range between 0 and 3. This scale has been designed to measure the frequency of anxiety symptoms experienced by the individual. The higher the total score, the higher the anxiety experienced by the person. Cronbach Alpha coefficient of the scale is 0.92. In this research, subject coefficient was found as 0.92 as well.

### 2.3 Procedure

Pre-test data was collected in one week by way of face-to-face interviews with the patients in the experimental and control groups, which interviews were held every day excluding Sunday. Information gathered by use of data collection tools was read and answers given were marked by the researcher. Likewise, post-test data was also collected in one week again by way of face-to-face interviews with the patients in the experimental and control groups, which interviews were held every day other than Sunday. Information gathered by means of data collection tools was read and answers given were marked by the researcher. Respective data was collected from the patients in the experimental and control groups two weeks after the completion of mindfulness-based stress reduction program that has been applied to the experimental group.

Criteria respecting inclusion in the research study;

• Being open to communication,
• Not suffering any diagnosed psychiatric disorder

Criteria respecting exclusion from the research study;

• Failure to participate in two applications minimum
• Failure to participate in post-tests

#### 2.3.1 Mindfulness-based stress reduction program

In this study, following the pre-test application, the experimental group was applied a mindfulness-based stress reduction program. The program has lasted 8 weeks and each session 2 h. Application of the program was conducted in the HD room. Due to inconvenient conditions prevailing at the hospital, MBSRP did not include yoga or meditation applications.

During the very first meeting with the patients in the experimental group, an acquaintance meeting was held which was followed by delivery of information on MBSR. In addition, a publication entitled “Mindfulness-Based Stress Reduction Program Training Booklet for Symptom Management in Hemodialysis Patients,” which was drawn up by the researcher by making use of the relevant literature, was distributed to the patients. Patients in the experimental group were divided into four subgroups on the basis of the day and time they would undergo HD, i.e., Monday-Wednesday-Friday morning and afternoon groups, and Tuesday-Thursday-Saturday morning and afternoon groups. The program was applied according to the following format: 2 days a week, 4 sessions a day, and one session a week for each group consisting of 10–15 patients.

In the course of the research process, no intervention was applied to the control group. Upon completion of the study, the “Mindfulness-Based Stress Reduction Program Training Booklet for Symptom Management in Hemodialysis Patients” was distributed to the patients in both groups (namely, experimental and control groups). The patients were recommended to properly and effectively do the exercises specified in the booklet concerned.

The researcher has participated in necessary certification programs to implement the MBSRP. Throughout the course of the program, patients in the experimental and control groups continued to undergo their pharmacological treatment, HD sessions, doctor checks, and routine applications of the HD center.

The contents of mindfulness-based stress reduction program are given in Table 1 below.

**TABLE 1:** The contents of mindfulness-based stress reduction program.

Week 1	Week 5
Acquaintance meeting Introducing mindfulness-based stress reduction program Sharing the experiences and expectations of individuals Doing mindful breathing exercise	Sharing experiences from the previous week Talking about mercy and forgiveness Doing mindful eating exercise
**Week 2**	**Week 6 (mindful silence day)**
Sharing experiences from the previous week Basics of communication, doing i language exercise Doing mindful listening exercise Distinguishing thought and awareness Ensuring that daily activities are conducted with mindfulness (eating food, brushing the teeth, taking shower)	Doing mindful silence exercise Achieving environmental mindfulness Sharing experiences in the end of mindful silence day
**Week 3**	**Week 7**
Sharing experiences from the previous week Doing mindful seeing exercise Doing abdominal/diaphragmatic breathing exercise	Sharing experiences from the previous week Communication exercises, achieving awareness in respect of communication language Doing relaxing/healing breathing exercise
**Week 4**	**Week 8**
Sharing experiences from the previous week Doing mindful observation exercise Noticing reactions to stress and ensuring that reactions are distinguished without any change Doing Tonglen (giving and taking) exercise	Sharing experiences from the previous week Identifying behaviors oriented to continuation of the exercises Determining methods that may be supported with a view to encouraging the continuation of the exercises

#### 2.3.2 Mindful breathing exercise

Create a comfortable posture. Sit with your spine erect and straight

Leave your eyes relaxed, they can be open or closed

Your hands are relaxed, close to your body, palms facing up or down

Breathe slowly, deeply

Bring your attention to the inflow and outflow of your breath

Follow the journey of your breath through your body, slowly watch how your nose, trachea and lungs react.

You may be distracted, focus on your breathing again

Try this for five minutes, then stop and evaluate how you feel.

#### 2.3.3 Mindful listening exercise

Mindful silence is more than just not speaking, it is an active silence. You can devote a day to this.

During active silence, you can eat and do your daily work.

Watching TV, listening to the radio, making phone calls and reading written materials should not be in silence.

Meanwhile, direct your attention to activated thoughts and emotions and stay aware.

Look at how you do your work during the silence, slow or fast?

Perceive what you felt and thought during the silence.

#### 2.3.4 Mindful seeing exercise

Create a comfortable posture. Sit with your spine erect and straight

Your eyes may be slightly closed

Breathe slowly, deeply and follow your breath

Bring your attention to your body. Perceive and accept what sensations you have in your body right now.

Then focus your attention on the sounds around you.

Detect low and loud sounds, detect whether sounds are close or far away.

Just perceive the sounds, you don’t need to give them meaning.

You may be distracted, turn to the sounds again

End the exercise and open your eyes slowly.

#### 2.3.5 Mindful observation exercise

For this exercise, you need a leaf or another object and 10–15 min of time.

Look at the object with all your attention and try to explore it

Touch the object, examine its color, shape, size and every detail. Look very closely and from a little distance. Smell it.

You may be distracted, try to bring your mind back.

End exercise

#### 2.3.6 Tonglen (giving and taking) exercise

Focus on your body when you see or think about a person you don’t like,

This doesn’t even have to be a person, it can be a memory that bothers you or a worry about the future.

If you feel tension in your body, first notice it,

Then allow it, don’t try to find a solution right away

After turning your face to this difficulty, take a breath in, inhale this difficulty, exhale and wish well.

These wishes may be wishes such as “I wish you to be at peace, I wish you to feel lighter, I wish you compassion, I wish you to be safe.”

#### 2.3.7 Mindful eating exercise

You can do this exercise with anything you eat or drink.

To practice the exercise, choose a certain meal (e.g., breakfast, lunch) or a certain food (e.g., bread, chocolate).

Let’s exemplify this exercise with tea.

Drink only tea, limit activities such as watching television, talking, reading.

Look at the color of the tea, smell it and perceive what its smell is.

Be thankful.

Think about where the tea grows and what stages it goes through until it reaches the cup.

Think about who provided it for you, who prepared it for you, and be grateful.

Close your eyes and sip your tea

Feel the feeling, taste and aroma of the tea in your mouth

Smell it, is it the same or different from what you first felt

Swallow slowly, paying attention to what you hear as it passes down your throat.

Pay attention to what you feel when swallowing

Think about the amount of sips you take, perceive how fast you drink, slow or fast.

Repeat these with every sip, wonder each time, try to understand again.

Don’t forget to smile

### 2.4 Statistical analysis of research data

In the analysis of data gathered during the research, percentage, frequency, chi-square analysis, *t*-test for independent groups, *t*-test for dependent groups were used by means of SPSS for Windows 22.00 statistical software package.

## 3 Findings

### 3.1 Comparison of hemodialysis patients in the experimental and control groups in terms of their defining characteristics

When defining characteristics of control and experimental groups as given in Table 2 are examined, a comparison made in respect of the patients’ ages, genders, educational background, employment status, level of income, presence of chronic diseases other than chronic renal failure (CRF), years of dialysis, and missed HD sessions, indicates that the experimental and control groups are quite alike (*p* > 0.05).

**TABLE 2:** Defining characteristics of HD patients in experimental and control groups.

		Experimental		Control		χ ^2^	*P*
		s	%	s	%		
Age	20 or younger	1	1.7	2	3.3	0.548	0.958
	21–40	6	10.0	7	11.7		
	41–60	19	31.7	20	33.3		
	61–80	30	50.0	28	46.7		
	81 or older	4	6.7	3	5.0		
Gender	Female	33	55.0	26	43.3	1.634	0.201
	Male	27	45.0	34	56.7		
Educational background	Illiterate	14	23.3	9	15.0	2.768	0.597
	Elementary	35	58.3	34	56.7		
	Junior High	2	3.3	4	6.7		
	High School	6	10.0	10	16.7		
	University	3	5.0	3	5.0		
Employed	Yes	5	8.3	4	6.7	0.120	0.729
	No	55	91.7	56	93.3		
Monthly income perception	Income < Expend.	20	33.3	12	20.0	2.780	0.249
	Income = Expend.	37	61.7	45	75.0		
	Income > Expend.	3	5.0	3	5.0		
Dialysis period (year)	0–1 year	4	6.7	7	11.7	3.024	0.388
	1–5 years	35	58.3	27	45.0		
	6–10 years	16	26.7	17	28.3		
	11 years or more	5	8.3	9	15.0		
Disease causing chronic renal failure	DM	22	36.7	25	41.7	1.549	0.818
	HT	18	30.0	17	28.3		
	Chronic pyelonephritis	0	0.0	1	1.7		
	Urological diseases	8	13.3	6	10.0		
	Other	12	20.0	11	18.3		
Any other chronic disease	Yes	44	73.3	43	71.7	0.042	0.838
	No	16	26.7	17	28.3		
Missed HD sessions	Yes	12	20.0	21	35.0	3.386	0.066
	No	48	80.0	39	65.0		

### 3.2 Frequency and mean value of symptoms experienced by HD patients in the experimental and control groups

When Table 3 is examined, it is observed that the highest complaints of the patients in the experimental group involved the following order of symptoms: fatigue or lethargy (80%), nervous temperament (75%), feeling sad (72.6%), distress (71.7%), sleep-maintenance insomnia (68.3%), sleep-onset insomnia (68.3%), numb or tingling feet (58.3%), and muscle cramps (58.1%). On the other hand, the lowest complaints of the patients in the experimental group included the following symptoms: poor concentration (20%), dry mouth (25%), coughing (26.7%), chest pain (28.3%), difficulty in keeping the legs still (31.7%), vomiting (33.3%), and diarrhea (33.3%).

**TABLE 3:** Distribution of the frequency and mean value of symptoms experienced by patients undergoing hemodialysis treatment.

Symptoms	Experimental		Control	
	%	Mean V.	%	Mean V.
Constipation	40.00	1.90	50.00	2.10
Nausea	38.30	1.75	31.70	1.68
Vomiting	33.30	1.58	28.30	1.53
Diarrhea	33.30	1.76	36.70	1.68
Poor appetite	43.30	1.93	58.30	2.13
Muscle cramps	58.10	2.43	40.00	1.76
Swollen legs	35.00	1.59	30.20	1.58
Shortness of breath	34.30	1.61	50.00	2.05
Drowsiness, dizziness	40.00	1.71	41.70	1.80
Difficulty in keeping the legs still	31.70	1.35	35.00	1.51
Numb or tingling feet	58.30	2.21	57.40	2.56
Fatigue or lethargy	80.00	2.65	75.00	2.91
Coughing	26.70	1.43	21.70	1.41
Dry mouth	25.00	1.25	21.70	1.55
Bone pain or arthralgia	35.00	1.40	48.30	2.26
Chest pain	28.30	1.30	20.70	1.20
Headache	38.30	1.93	57.50	2.58
Muscle pain	38.30	1.50	30.10	1.63
Poor concentration	20.00	1.26	38.40	1.60
Skin dryness	51.70	1.98	45.20	1.78
Itching, rashes	51.70	2.10	52.10	2.23
Distress	71.70	2.25	74.30	2.55
Nervous temperament	75.00	2.53	68.50	2.41
Feel sad	72.60	2.28	71.70	2.60
Sleep-maintenance insomnia	68.30	2.60	58.90	2.43
Feel uncomfortable	58.30	1.85	68.50	2.10
Sleep-onset insomnia	68.30	2.61	58.90	2.41
Feel anxious	58.00	2.16	68.50	2.15
Reduced sexual interest	45.00	1.96	56.90	2.23
Reduced sexual satisfaction	45.00	1.96	56.90	2.23

According to Table 3, patients in the control group complained about the following symptoms for the most part: fatigue or lethargy (75.0%), distress (74.3%), feeling sad (71.7%), nervous temperament (68.5%), sleep-onset insomnia (58.9%), sleep-maintenance insomnia (58.9%), headache (57.5%), and numb or tingling feet (57.4%). On the other hand, the lowest complaints of the patients in the control group involved the following symptoms: chest pain (20.7%), dry mouth (21.7%), coughing (21.7%), muscle pain (30.1%), swollen legs (30.2%), difficulty in keeping the legs still (35.0%), and poor concentration (38.4%).

### 3.3 Comparison of pre-tests and post-tests of the experimental group before and after the trainings

When Table 4 is examined, it is observed that the *t*-test analyses of the differences between PSS, BAS, MAAS and DSI pre-test and post-test scores of HD patients in the experimental group before and after the trainings provided were found to be significant in favor of the post-tests (*p* < 0.001, *p* < 0.01).

**TABLE 4:** Comparison of pre-test and post-test scores of HD patients in the experimental group before and after the trainings.

		$\bar{X}$ ± S.s.	*t*	*p*
Perceived stress scale (PSS)	Pre-test	24.75 ± 3.50	3.478	0.001
	Post-test	22.17 ± 4.322		
Beck anxiety scale (BAS)	Pre-test	34.92 ± 7.041	3.800	0.000
	Post-test	30.40 ± 5.863		
Mindful attention awareness scale (MAAS)	Pre-test	41.77 ± 10.96	−6.941	0.000
	Post-test	54.78 ± 9.395		
Dialysis symptom index (DSI)	Pre-test	60.73 ± 16.58	3.261	0.002
	Post-test	51.98 ± 11.967		

### 3.4 Comparison of pre- and post-tests of hemodialysis patients in the control group before and after the trainings

When Table 5 is examined, it was found that the differences between the BAS pre-test and post-test scores of HD patients in the control group before and after the study were significant against the post-tests, while the differences between the PSS, MAAS, DSI pre-test and post-test scores were statistically insignificant.

**TABLE 5:** Comparison of pre-test and post-test scores of hemodialysis patients in the control group before and after the trainings.

		$\bar{X}$ ± S.s.	*t*	*p*
Perceived stress scale (PSS)	Pre-test	24.60 ± 4.37	−1.293	0.201
	Post-test	25.68 ± 4.69		
Beck anxiety scale (BAS)	Pre-test	33.45 ± 7.16	−3.693	0.000
	Post-test	39.62 ± 10.50		
Mindful attention awareness scale (MAAS)	Pre-test	43.95 ± 4.22	1.260	0.213
	Post-test	41.82 ± 11.32		
Dialysis symptom index (DSI)	Pre-test	56.90 ± 14.11	−0.718	0.476
	Post-test	58.67 ± 15.89		

According to research data, it may be suggested that MBSRP was effective on PSS, BAS, MAAS and DSI scores, i.e., the MBSRP applied has reduced stress and anxiety levels of hemodialysis patients and raised their conscious mindfulness and symptom management levels.

## 4 Discussion

The findings and results of the research that was conducted with a view to determining the effect of conscious mindfulness based informative approaches applied in the case of HD patients on reducing stress and managing symptoms have been discussed in line with the relevant literature. As a result of the literature research and review, it has been observed that the process involving application of mindfulness-based stress reduction program to HD patients was quite new wherefore only a limited number of studies could be accessed. For this reason, research results were discussed in reference to the findings of the most recent study as well.

According to the results of our study it is observed that the highest complaints of the patients in the experimental group involved the following symptoms: fatigue or lethargy, nervous temperament, feeling sad, distress, sleep-maintenance insomnia, sleep-onset insomnia, numb or tingling feet, and muscle cramps. On the other hand, the lowest complaints of the patients in the experimental group included the following symptoms: poor concentration, dry mouth, coughing, chest pain, difficulty in keeping the legs still, vomiting, and diarrhea. In the case of control group, patients complained about the following symptoms for the most part: fatigue or lethargy, distress, feeling sad, nervous temperament, sleep-onset insomnia, sleep-maintenance insomnia, headache, and numb or tingling feet. On the other hand, the lowest complaints of the patients in the control group involved the following symptoms: chest pain, dry mouth, coughing, muscle pain, swollen legs, difficulty in keeping the legs still, and poor concentration.

In the study by Hintistan and Deniz (2018) that was conducted with respect to Symptom Evaluation in Patients Undergoing HD Treatment, most frequent symptoms experienced by HD patients were identified as fatigue/lethargy, bone pain/arthralgia, and muscle cramps. According to a research by Taylan and Özkan (2020), symptom clusters were grouped as follows: feeling sad, nervous temperament, distress, feeling anxious, and other similar psychological symptoms as the first group; sleep-onset insomnia, sleep-maintenance insomnia, feeling uncomfortable, and shortness of breath symptoms as the second group; and reduced sexual interest and reduced sexual satisfaction symptoms as the third group. On the other hand, in a study by Thong et al. (2009), symptom clusters were divided into the following groups: shortness of breath, drowsiness, and poor appetite as the first group; muscle pain, chest pain, and numb hands/feet as the second group; and skin dryness and itching/rashes as the third group.

When the literature is examined, it is observed that, in terms of symptoms -which the HD patients complained about- with the highest and lowest frequencies, findings in the literature are similar to those of our research in some cases but also differ from our findings in some other cases. It is considered that such difference stems from adherence/nonadherence to treatment and diet program, presence of other chronic diseases, and medications administered.

In our study, it was found that MBSRP reduced the level of stress and anxiety experienced by hemodialysis patients and enhanced their levels of conscious mindfulness and symptom management.

Additionally, in our study, it was observed that there was a negative change in the PSS, BAS, MAAS and DSI scores of HD patients in the control group.

In their study aimed at determining the effectiveness of mindfulness-based stress reduction program in HD patients experiencing anxiety and depression, Haghshenas et al. (2019) have shown that MBSRP reduced the level of anxiety and depression suffered by such HD patients. In a research by Solati et al. (2019) which was conducted with respect to the effect of Mindfulness-Based Cognitive Therapy on the life quality and self-sufficiency/efficacy of HD patients, it was determined that mindfulness-based cognitive therapy program has enhanced the quality of life and self-sufficiency/efficacy levels of such HD patients.

On the other hand, in a study by Sohn et al. (2018) that was undertaken for the purpose of identifying the effectiveness of mindfulness and group cognitive behavioral therapy in HD patients experiencing end-stage renal failure, it was determined that Cognitive Behavioral Therapies involving mindfulness-based cognitive therapy program (MBCTP) and mindfulness-based stress reduction program (MBSRP) have improved general mental health and biochemical marker levels of individuals with end-stage renal failure who have been undergoing hemodialysis. In their research which was conducted with a view to evaluating the impact of mindfulness program on the general health of patients undergoing hemodialysis, Nejad et al. (2018) have expressed that mindfulness was effective in reducing physical symptoms and anxiety, sleep disorders, social malfunctions, and depression symptoms.

According to the study conducted by Razzera et al. (2022), to investigate the effect of mindfulness-based programs on chronic renal failure patients receiving hemodialysis treatment; mindfulness-based programs can offer a promising and safe complementary therapy for people with CRF undergoing hemodialysis, acting on quality of life and physical aspects of the disease. According to the study conducted by Nassim et al. (2021), in patients undergoing dialysis, mindfulness-based programs may be helpful intervention for depression symptom. Alhawatmeh et al. (2022) in his studies (2022); found that mindfulness meditation is effective in managing stress and improving quality of life in patients undergoing hemodialysis.

According to Carver and Cheung (2021); yoga breathing/mindfulness meditation on symptoms and COVID-19-related anxiety in patients receiving dialysis is feasible and acceptable. According to Rigas et al. (2022); mindfulness-based interventions is effective in treating depression and anxiety and improving mood disorder symptoms in patients receiving hemodialysis.

In their study, Al-Ghabeesh et al. (2021) found that mindfulness could improve the psychological health of hemodialysis patients. According to Garel et al. (2023); mindfulness-based stress reduction (MBSR) intervention is effective in managing pain and psychiatric symptoms in hemodialysis patients. According to Hernandez et al. (2021); Awareness-Based Program in Hemodialysis Patients reduces symptom severity without negative effects. According to the study of Dehghan et al. (2021); Awareness is effective in reducing coronavirus anxiety in hemodialysis patients.

When results derived from studies applied in the case of HD patients are examined, it is observed that such findings are similar to those of our research.

In their relevant study, Shapiro et al. (2005) have reported that, as a result of mindfulness-based stress reduction program for healthcare professionals, the individuals who took part in the program stated considerable reduction in perceived stress, increase in self-compassion, more content with life, lower job burnout, and diminished distress. In the research undertaken by Xunlin et al. (2020) with a view to determining the effectiveness of mindfulness-based interventions in the case of cancer patients, it was identified that mindfulness-based interventions might be used as an adjuvant therapy in the treatment of symptoms associated with cancer. On the other hand, Medina et al. (2017) have specified in their study on the effect of mindfulness on DM that mindful awareness may exert positive influences on any and all aspects of diabetes.

In a study by Ozdemir and Kavak Budak (2022) it has been determined that considerable enhancement was registered with respect to the level of hope, psychological wellbeing and functional healing, and that mindfulness-based stress reduction therapy was more effective than psychoeducation in raising the level of hope, psychological wellbeing and functional healing of schizophrenic patients. On the other hand, Sarıtas and Aktura (2020) have identified in their research that mindful awareness of the patients with cardiac insufficiency was at medium level, and that conscious mindfulness affected the level of anxiety and depression positively.

In her study, Sener and Timur Tashan (2021) has determined that mindfulness-based stress reduction program was effective in reducing menopausal complaints of women in the postmenopausal period and also in enhancing their quality of life in the subject period. According to a research by Heide et al. (2021) it was concluded that mindfulness had strong effects on anxiety and depressed mood of Parkinson patients, and that mindfulness might reduce the intensity of symptoms thereby improving the general condition of the patient. According to Kerr et al. (2013) study, mindfulness-based practices reduce stress in chronic pain and the risk of recurrence of depression. According to the same study, mindfulness-based practices increase cognitive regulation and thus increase metacognition.

Gherardi-Donato et al. (2023) in their study, they showed that the mindfulness-based program was effective on mental health psychometric measures (perceived stress and anxiety) and long-term stress biomarkers (hair cortisol). According to the study, the mindfulness program is also effective in improving the mental health of university employees. Finally, in a study by Gross et al. (2010) it was determined that MBSRP reduced anxiety, depression and bothersome symptoms of poor sleep in the case of transplant recipients, and hence enhanced their quality of life. According to Guu et al. (2023); mindfulness-based stress reduction program (MBSR) affects brain functionality.

As a result of the literature research and review, it was observed that conscious mindfulness-based informative approaches applied in groups suffering chronic diseases other than CRF and in non-patient groups brought about positive changes.

## 5 Conclusion

In this research which was conducted so as to determine the effect of conscious mindfulness-based informative approaches applied in hemodialysis patients on reducing stress and managing symptoms, it was found out that the conscious mindfulness-based informative approaches decreased the perceived stress and anxiety of HD patients, whereas increased their levels of conscious mindfulness and symptom management.

## 6 Ethical aspect of the research

Necessary research ethics committee approval was obtained from İnönü University Scientific Research and Publication Ethics Board and Malatya Clinical Research Ethics Board. In addition, verbal and written permits were received from Atatürk University Research Hospital and Erzurum Regional Training and Research Hospital. With a view to implementing the MBSR program, the researcher has participated in necessary certification programs. Throughout the course of the program, patients in the experimental and control groups continued to undergo their pharmacological treatment, HD sessions, doctor checks, and routine applications of the HD center.

## 7 Limitations, future directions and implications

The strength of this study is that there are a limited number of studies examining the effect of MBSRP use on hemodialysis patients. In recent years, non-pharmacological complementary and integrative interventions have become increasingly used in the management of diseases. Among these initiatives, awareness-based interventions have begun to be increasingly implemented in families, the elderly, children, women, and various patient groups.

Applications such as MBSRP are a powerful tool in improving the relationship between healthcare professionals and patients, decision-making, diagnosis, therapeutic approach, and reducing stress in the patient and healthcare professionals themselves. Considering that the responsibilities of health professionals will increase over time, it is seen that individuals need to follow and be informed about both pharmacological and non-pharmacological applications for their problems. In order to be beneficial to themselves and individuals, healthcare professionals must know both the psychological and biological effects of MBSRP and complete the necessary certification and training programs to implement the program more effectively.

The limitation of this study is that the duration of monitoring individuals was limited to 8 weeks because the program was carried out within a certain period of time. Research results can be generalized to groups and the population that are similar in terms of inclusion criteria and study variables. In line with these results, it is recommended that conscious mindfulness-based informative approaches such as mindfulness-based cognitive therapy program and mindfulness-based stress reduction program be added to treatment and care programs of hemodialysis patients, and that new studies be undertaken to ensure the comparison of different complementary and supportive alternative medicine applications with mindfulness-based stress reduction program.

## Author’s note

This study was produced from the doctoral thesis of the first author.

## Data availability statement

The original contributions presented in this study are included in this article/supplementary material, further inquiries can be directed to the corresponding author.

## Ethics statement

The studies involving humans were approved by the İnönü University Scientific Research and Publication Ethics Board and Malatya Clinical Research Ethics Board. The studies were conducted in accordance with the local legislation and institutional requirements. The participants provided their written informed consent to participate in this study.

## Author contributions

GA: Conceptualization, Visualization, Writing – original draft, Writing – review & editing. BE: Formal analysis, Methodology, Project administration, Writing – original draft, Writing – review & editing.

## References

[B1] Akgöz N. Arslan S. (2017). Examination of symptoms experienced in patients receiving hemodialysis treatment. *Turk. Nephrol. Dial. Transplant. Nurses Assoc. Nephrol. Nurs. J.* 1 20–28.

[B2] Al-Ghabeesh S. H. Rayan A. Hattab F. Jarrar Y. (2021). Mindfulness and psychological distress among hemodialysis patients. *Psychol. Health Med.* 27 917–924. 10.1080/13548506.2021.1960395 34320891

[B3] Alhawatmeh H. Alshammari S. Rababah J. A. (2022). Effects of mindfulness meditation on trait mindfulness, perceived stress, emotion regulation, and quality of life in hemodialysis patients: A randomized controlled trial. *Int. J. Nurs Sci.* 9 139–146. 10.1016/j.ijnss.2022.03.004 35509694 PMC9052255

[B4] Almutary H. (2022). Depression, sleep disturbance, and quality of life in patients undergoing dialysis therapy. *Appl. Nurs. Res.* 67:151610. 10.1016/j.apnr.2022.151610 36116860

[B5] Al-Nashri F. Almutary H. (2022). Impact of anxiety and depression on the quality of life of haemodialysis patients. *J. Clin. Nurs.* 31 220–230. 10.1111/jocn.15900 34114273

[B6] Beck A. T. Epstein N. Brown G. Steer R. A. (1988). An inventory for measuring clinical anxiety: Psychometric properties. *J. Consult. Clin. Psychol*. 56, 893–897. 3204199 10.1037//0022-006x.56.6.893

[B7] Boyd J. E. Lanius R. A. McKinnon M. C. (2018). Mindfulness-based treatments for posttraumatic stress disorder: A review of the treatment literature and neurobiological evidence. *J. Psychiatry Neurosci.* 43 7–25. 10.1503/jpn.170021 29252162 PMC5747539

[B8] Brown K. W. Ryan R. M. (2003). The benefits of being present: Mindfulness and its role in psychological well-being. *J. Pers. Social Psychol*. 84, 822–848. 10.1037/0022-3514.84.4.822 12703651

[B9] Carver J. A. Cheung K. L. (2021). Feasibility and acceptability of a yogic breathing/mindfulness meditation e-intervention on symptoms and COVID-19-associated anxiety in patients receiving dialysis. *J. Palliat. Med.* 24 1124–1125. 10.1089/jpm.2021.0161 34152865 PMC10039269

[B10] Catak P. D. Ogel K. (2010). Awareness based therapies and therapeutic processes. *Clin. Psychiatry* 13 85–91.

[B11] Dehghan M. Namjoo Z. Mohammadi, Akbarabadi F. Fooladi Z. Zakeri M. A. (2021). The relationship between anxiety, stress, spiritual health, and mindfulness among patients undergoing hemodialysis: A survey during the COVID-19 outbreak in Southeast Iran. *Health Sci. Rep.* 4:e461. 10.1002/hsr2.461 34938901 PMC8670730

[B12] Eren G. (2019). *Evaluation of symptoms and quality of life in patients receiving hemodialysis treatment. Institute of health sciences, department of internal medicine nursing.* Manisa: Manisa Celal Bayar University.

[B13] Eskin M. Demirkıran F. Harlak H. Dereboy Ç (2013). The adaptation of the perceived stress scale into Turkish: A reliability and validity analysis. *New Symp. J.* 51 132–140.

[B14] Garel N. Rigas C. Ben M’rad M. Bodenstein K. Joober R. Sekhon H. (2023). Mindfulness-based intervention for benzodiazepine deprescription in hemodialysis patients with anxiety and depressive symptoms. *J. Psychiatry Neurosci.* 48 E149–E150. 10.1503/jpn.220216 37172961 PMC10185346

[B15] Gherardi-Donato E. C. D. S. Gimenez L. B. H. Fernandes M. N. F. Lacchini R. Júnior C. E. B. Díaz-Serrano K. V. (2023). Mindfulness practice reduces hair cortisol, anxiety and perceived stress in university workers: Randomized clinical trial. *Healthcare* 11:2875. 10.3390/healthcare11212875 37958019 PMC10648523

[B16] Gross C. R. Kreitzer M. J. Thomas W. Reilly-Spong M. Cramer-Bornemann M. Nyman J. A. (2010). Mindfulness-based stress reduction for solid organ transplant recipients: A randomized controlled trial. *Altern. Ther. Health Med.* 16 30–38. PMC3076132 20882729

[B17] Guu S. F. Chao Y. P. Huang F. Y. Cheng Y. T. Hydra, Ng H. Y. (2023). Interoceptive awareness: MBSR training alters information processing of salience network. *Front. Behav. Neurosci.* 17:1008086. 10.3389/fnbeh.2023.1008086 37025109 PMC10070746

[B18] Haghshenas M. Assarian F. Omidi A. Razaghof M. (2019). Efficacy of mindfulness-based stress reduction in hemodialysis patients with anxiety and depression: A randomized, double-blind, parallel-group trial. *Ephysician* 11 7370–7377. 10.19082/7370

[B19] Harvard Business School Publishing and Corporation (2017). Mindfulness. *Harvard Bus. Rev. Press* 473 1–127.

[B20] Heide A. Speckens A. Meinders M. J. Rosenthal L. S. Bloem B. R. Helmich R. C. (2021). Stress and mindfulness in Parkinson’s disease – a survey in 5000 patients. *NPJ Parkinsons Dis.* 7:7. 10.1038/s41531-020-00152-9 33462213 PMC7813889

[B21] Hernandez R. Burrows B. Browning M. H. E. M. Solai K. Fast D. Litbarg N. O. (2021). Mindfulness-based virtual reality intervention in hemodialysis patients: A pilot study on end-user perceptions and safety. *Kidney360* 2 435–444. 10.34067/KID.0005522020 35369024 PMC8786010

[B22] Hintistan S. Deniz A. (2018). Evaluation of symptoms in patients undergoing hemodialysis. *Bezmialem Sci.* 6 112–118. 10.14235/bs.2018.1530

[B23] Igarashi N. S. Karam C. H. Afonso R. F. Carneiro F. D. Lacerda S. S. Santos B. F. (2021). The effects of a short-term meditation-based mindfulness protocol in patients receiving hemodialysis. *Psychol. Health Med.* 15 1–10. 10.1080/13548506.2021.1871769 33449820

[B24] Ji C. F. Wu G. H. Du X. D. Wang G. X. Liu L. L. Niu M. E. (2023). Factors that contribute to trait mindfulness level among hospitalized patients with major depressive disorder. *Front. Psychiatry* 29:1144989. 10.3389/fpsyt.2023.1144989 37496685 PMC10368243

[B25] Kabat-Zinn J. (2003). Mindfulness-based interventions in context: Past, present, and future. *Clin. Psychol.* 10 144–156. 10.1093/clipsy.bpg016

[B26] Kerr C. E. Sacchet M. D. Lazar S. W. Moore C. I Jone S. R. (2013). Mindfulness starts with the body: Somatosensory attention and top-down modulation of cortical alpha rhythms in mindfulness meditation. *Front. Hum. Neurosci.* 13:12. 10.3389/fnhum.2013.00012 23408771 PMC3570934

[B27] Körükcü Ö Kukulu K. (2015). A program to protect body-mind-spirit integrity: Awareness-based stress reduction program. *Curr. Approach. Psychiatry* 7 68–74. 10.5455/cap.20140504031811

[B28] Mantzios M. Giannou K. (2018). When did coloring books become mindful? Exploring the effectiveness of a novel method of mindfulness-guided instructions for coloring books to increase mindfulness and decrease anxiety. *Front. Psychol.* 30:56. 10.3389/fpsyg.2018.00056 29441038 PMC5797627

[B29] Medina W. L. Wilson D. Salvo V. Vannucchi B. Souza ÉL. Lucena L. (2017). Effects of mindfulness on diabetes mellitus: Rationale and overview. *Curr. Diabetes Rev.* 13 141–147. 10.2174/1573399812666160607074817 27280721

[B30] Mertens D. M. (2019). *Integrating research and evaluation diversity in education and psychology with quantitative, qualitative and mixed methods.* Ankara: Anı Publishing.

[B31] Nassim M. Park H. Dikaios E. Potes A. Elbaz S. Mc Veigh C. (2021). Brief mindfulness intervention vs. health enhancement program for patients undergoing dialysis: A randomized controlled trial. *Healthcare* 9:659. 10.3390/healthcare9060659 34205915 PMC8228217

[B32] Nejad M. M. Shahgholian N. Samouei R. (2018). The effect of mindfulness program on general health of patients undergoing hemodialysis. *J. Educ. Health Promot.* 7:74. 10.4103/jehp.jehp_132_17 29963567 PMC6009148

[B33] Onsöz H. B. Yesilbalkan ÖU. (2013). Reliability and validity of the turkish version of the dialysis symptom ındex in chronic hemodialysis patients. *Turk. Neph. Dial. Transplant.* 22 60–67. 10.5262/tndt.2013.1001.08

[B34] Ozdemir A. A. Kavak Budak F. (2022). The effects of mindfulness-based stress reduction training on hope, psychological well-being, and functional recovery in patients with schizophrenia. *Clin. Nurs. Res.* 31 183–193. 10.1177/10547738211039069 34382427

[B35] Ozyeşil Z. Arslan C. Kesici S. Deniz M. N. (2011). Adaptation of the mindful attention awareness scale into Turkish. *Educ. Sci.* 36 224–235.

[B36] Razzera B. N. Adamoli A. N. Ranheiri M. F. Oliveira M. D. S. Feoli A. M. P. (2022). Impacts of mindfulness-based interventions in people undergoing hemodialysis: A systematic review. *J. Bras. Nefrol.* 44 84–96. 10.1590/2175-8239-JBN-2021-0116 34643641 PMC8943880

[B37] Rigas C. Park H. Nassim M. Su C. L. Greenway K. Lipman M. (2022). Long-term effects of a brief mindfulness intervention versus a health enhancement program for treating depression and anxiety in patients undergoing hemodialysis: A randomized controlled trial. *Can. J. Kidney Health Dis.* 9:20543581221074562. 10.1177/20543581221074562 35273807 PMC8902179

[B38] Sanlıtürk D. Ovayolu N. Kes D. (2018). Common problems and solution suggestions in hemodialysis patients. *Turk. Nephrol. Dial. Transplant. Nurses Assoc. Nephrol. Nurs. J.* 1 17–25.

[B39] Sarıtas S. Ç Aktura S. Ç (2020). Does mindfulness affect the hospital anxiety-depression level of patients with heart failure? *J. Cardiovasc. Nurs.* 11 1–6.

[B40] Sener N. Timur Tashan S. (2021). The effects of mindfulness stress reduction program on postmenopausal women’s menopausal complaints and their life quality. *Complement. Ther. Clin. Pract.* 45:101478. 10.1016/j.ctcp.2021.101478 34543872

[B41] Shapiro S. L. Astin J. A. Bishop S. R. Cordova M. (2005). Mindfulness-based stress reduction for health care professionals: Results from a andomized trial. *Int. J. Stress Manag.* 12 164–176. 10.1037/1072-5245.12.2.164

[B42] Sohn B. K. Oh Y. K. Choi J. S. Song J. Lim A. Lee J. P. (2018). Effectiveness of group cognitive behavioral therapywith mindfulness in end-stage renal disease hemodialysis patients. *Kidney Res. Clin. Pract.* 37 77–84. 10.23876/j.krcp.2018.37.1.77 29629280 PMC5875579

[B43] Solati K. Mardani S. Ahmadi A. Danaei S. (2019). Effect of mindfulness-based cognitive therapy on quality of life and self-efficacy in dialysis patients. *J. Renal. Inj. Prev.* 8 28–33. 10.15171/jrip.2018.06

[B44] Taylan S. Özkan I. (2020). Relationship between symptom clusters and sexual function in hemodialysis patients. *J. Nephrol. Nurs.* 15 91–100. 10.1111/jorc.12051 24438743

[B45] Thomas Z. Novak M. Platas S. G. T. Gautier M. Holgin A. P. Fox R. (2017). Brief mindfulness meditation for depression and anxiety symptoms in patients undergoing hemodialysis. *Clin. J. Am. Soc. Nephrol.* 12 2008–2015. 10.2215/CJN.03900417 29025788 PMC5718270

[B46] Thong M. S. Y. Dijk S. V. Noordzij M. Boeschoten E. W. Krediet R. T. Dekker F. W. (2009). Symptom clusters in incident dialysis patients: Associations with clinical variables and quality of life. *Nephrol. Dial. Transplant.* 24 225–230. 10.1093/ndt/gfn449 18689791

[B47] Tırıskan M. Onnar N. Çetin Y. A. Cömert I. T. (2015). The importance of conscious awareness in preventing relapse in substance addiction: A review study. *Turk. J. Addict.* 2 123–142.

[B48] Tolasa A. G. Akyol A. (2017). Use of aromatherapy in dialysis patients. *Turk. Nephrol. Dial. Transplant. Nurses Assoc. Nephrol. Nurs. J.* 2 1–7.

[B49] Tuot D. S. Velasquez A. McCulloch C. E. Banerjee T. Zhu Y. Hsu C. (2015). The kidney awareness registry and education (KARE) study: Protocol of a randomized controlled trial to enhance provider and patient engagement with chronic kidney disease. *BMC Nephrol.* 16:166. 10.1186/s12882-015-0168-4 26494562 PMC4618520

[B50] Ulusoy M. Şahin N. H. Erkmen H. (1998). Turkish version of the anxiety inventory: Psychometric properties. *J. Cogn. Psychother.* 12 163–172.

[B51] Weisbord S. D. Fried L. F. Arnold R. M. Rotondi A. J. Fine M. J. Levenson D. J. (2004). Development of a symptom assessment instrument for chronic hemodialysis patients: The Dialysis Symptom Index. *J. Pain Symptom Manage*. 27, 226–240. 10.1016/j.jpainsymman.2003.07.004 15010101

[B52] Xunlin N. G. Lau Y. Klainin-Yobas P. (2020). The effectiveness of mindfulness-based interventions among cancer patients and survivors: A systematic review and meta-analysis. *Support Care Cancer* 28 1563–1578. 10.1007/s00520-019-05219-9 31834518

